# The complete plastid genome of *Holcoglossum singchianum* (Orchidaceae, Vandeae)

**DOI:** 10.1080/23802359.2019.1704185

**Published:** 2020-01-21

**Authors:** Jin-Liao Chen, Xiong-De Tu, Ding-Kun Liu, Sai Zhang, Ming-He Li

**Affiliations:** a Key Laboratory of National Forestry and Grassland Administration for Orchid Conservation and Utilization at College of Landscape Architecture, Fujian Agriculture and Forestry University, Fuzhou, PR China;; b Fujian Colleges and Universities Engineering Research Institute of Conservation and Utilization of Natural Bioresources at College of Forestry, Fujian Agriculture and Forestry University, Fuzhou, PR China

**Keywords:** Chloroplast genome, *Holcoglossum*, phylogeny, Vanda clade

## Abstract

The complete plastid genome of *Holcoglossum singchianum* was determined and analyzed in this work. The plastome was 147,715 bp in length with 84,094 bp of the large single-copy (LSC) region, 12,073 bp of the small single-copy (SSC) region and 25,774 bp of the inverted repeat (IRs) regions. The genome contained 120 genes, 74 protein-coding genes, 38 tRNA genes, and 8 rRNA genes. Phylogenetic analysis of 20 Aeridinae plastomes suggested three groups of *Holcoglossum* were divided, and *H. singchianum* was sister to *H. lingulatum*.

The genus *Holcoglossum* (Aeridinae, Orchidaceae) described by Schlechter (1919) based on *Saccolabium quasipinifolium*, comprises about 20 species that are mainly distributed in southwestern China and neighboring regions (Zhang et al. 2013; Pridgeon et al. 2014). *Holcoglossum* has two diversity hotspots, the tropical region and temperate alpine region of the Hengduan Mountains (Pridgeon et al. 2014). The genus is characterized by the horn-shaped spur, porate pollinia directly attached to a common stipe, and white lip (Zhang et al. 2013). *Holcoglossum singchianum* was found in Yunnan Province and described by Zhang et al. (2013).

Fresh leaf sample of *H. singchianum* was acquired from Xichou County, Yunnan Province of China (23°25′N, 104°41′E). The voucher specimen was deposited at Fujian Agriculture and Forestry University (specimen code MH Li or080). DNA extraction, library constructing, sequencing, and data filtering were referenced in Liu et al. (2019). The plastid genome of *H. wangii* (MK442935) as reference, the paired-end reads were filtered with GetOrganelle pipe-line (Jin et al. 2018) to get plastid-like reads, then the filtered reads were assembled by SPAdes version 3.10 (Bankevich et al. 2012), the final ‘fastg’ were filtered by the script of GetOrganelle to get pure plastid contigs, and the filtered De Brujin graphs were viewed and edited by Bandage (Wick et al. 2015). Assembled plastid genome annotation based on comparison with the plastome of *H. wangii* by GENEIOUS version 11.1.5 (Biomatters Ltd., Auckland, New Zealand) (Kearse et al. 2012). The matrix of 20 representative species of Aeridinae and were aligned using MAFFT version 7.307 (Katoh and Standley 2013). The phylogenetic tree was constructed based on the complete plastid genomes by the maximum likelihood software IQ-TREE (Nguyen et al. 2015) and branch supports with the ultrafast bootstrap (Hoang et al. 2018).

The complete plastid genome sequence of *H. singchianum* (GenBank accession MN732560) was 147,715 bp in length, with a large single-copy (LSC) region of 84,094 bp, a small single-copy (SSC) region of 12,073 bp, and a pair of inverted repeats (IRs) regions of 25,774 bp. The complete genome GC content was 36.7% (LSC, 34.0%; SSC, 27.9%; IR, 43.1%) and the plastome contained 120 genes, 74 protein-coding genes, 38 tRNA genes, and 8 rRNA genes.

The phylogenetic analysis of 20 Aeridinae plastomes showed that *H. singchianum* was sister to *H. lingulatum* and two groups, *Holcoglossum* and *Vanda*, were divided with full support (Figure 1). The 13 species of *Holcoglossum* were subdivided into three groups.

**Figure 1.**
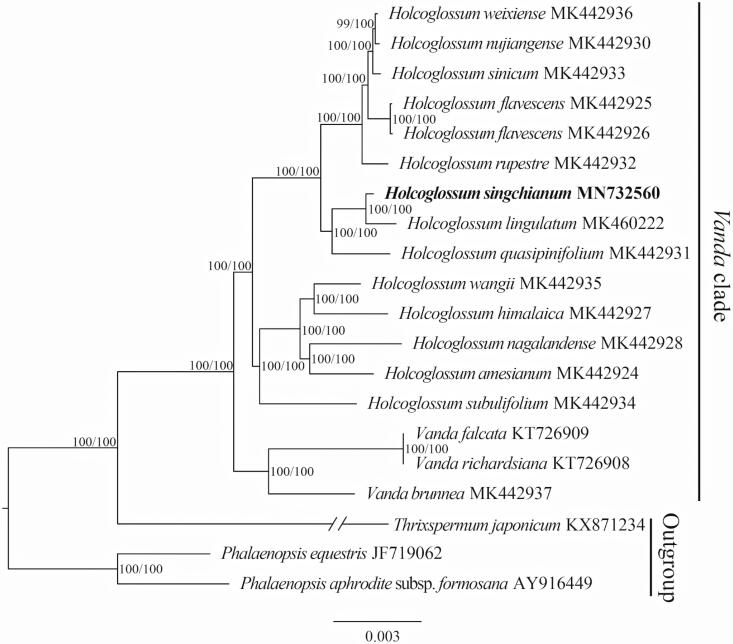
The maximum-likelihood (ML) tree based on the 20 plastid genomes of Aeridinae. Numbers near the nodes mean bootstrap support value (Standard bootstrap left and Ultrafast bootstrap right).
